# Anti-Cancer Agent: The Labdane Diterpenoid-Andrographolide

**DOI:** 10.3390/plants12101969

**Published:** 2023-05-12

**Authors:** Rosa Tundis, Jayanta Kumar Patra, Marco Bonesi, Subrata Das, Rajat Nath, Anupam Das Talukdar, Gitishree Das, Monica Rosa Loizzo

**Affiliations:** 1 Department of Pharmacy, Health and Nutritional Sciences, University of Calabria, 87036 Rende, Italy; rosa.tundis@unical.it (R.T.);; 2 Research Institute of Integrative Life Sciences, Dongguk University-Seoul, Goyangsi 10326, Republic of Korea; jkpatra@dongguk.edu; 3 Department of Botany and Biotechnology, Karimganj College, Assam University, Assam 788710, India; 4 Department of Life Science and Bioinformatics, Assam University, Assam 788011, India

**Keywords:** cancer, mechanism of action, apoptosis, autophagy, angiogenesis, enhanced radio-sensitivity

## Abstract

In spite of the progress in treatment strategies, cancer remains a major cause of death worldwide. Therefore, the main challenge should be the early diagnosis of cancer and the design of an optimal therapeutic strategy to increase the patient’s life expectancy as well as the continuation of the search for increasingly active and selective molecules for the treatment of different forms of cancer. In the recent decades, research in the field of natural compounds has increasingly shifted towards advanced and molecular level understandings, thus leading to the development of potent anti-cancer agents. Among them is the diterpene lactone andrographolide, isolated from *Andrographis paniculata* (Burm.f.) Wall. ex Nees that showed shows a plethora of biological activities, including not only anti-cancer activity, but also anti-inflammatory, anti-viral, anti-bacterial, neuroprotective, hepatoprotective, hypoglycemic, and immunomodulatory properties. Andrographolide has been shown to act as an anti-tumor drug by affecting specific molecular targets that play a part in the development and progression of several cancer types including breast, lung, colon, renal, and cervical cancer, as well as leukemia and hepatocarcinoma. This review comprehensively and systematically summarized the current research on the potential anti-cancer properties of andrographolide highlighting its mechanisms of action, pharmacokinetics, and potential side effects and discussing the future perspectives, challenges, and limitations of use.

## 1. Introduction

In spite of the progress of treatment strategies, cancer remains a major cause of death worldwide [1]. The conventional cancer treatments include chemotherapy, radiotherapy, and surgical removal. However, in some cases the resistance of cells to these therapies reduces their effectiveness. The incidence of cancer and the mortality rate have risen exponentially, with about 19.3 million new cancer cases in 2020 [2]. Despite the advances of cancer treatments, that include surgery, conventional chemotherapy, radiation therapy, hormone therapy, and immunotherapy, the overall disease-free survival rate is still inadequate. Additionally, the toxicity often associated with anti-cancer drug therapy poses additional challenges. Therefore, the search for non-toxic alternative therapies, including the use of non-toxic natural compounds of plant origin, for the prevention and treatment of cancer is drawing increasing attention. Due to their availability and wide margin of safety, plant-derived products have made a great impact on drug discovery and are gaining increasing attention for both cancer prevention and treatment [1,3].

**Figure 1:**
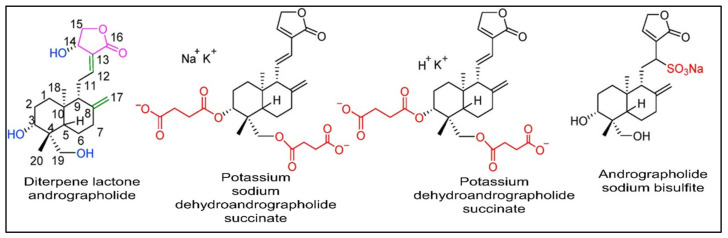
The chemical configuration of andrographolide and its derivatives that are used commercially in clinical practices. Reproduced with permission from Zhang et al. [21], licensed content publisher—Elsevier.

Generally, natural compounds display multi-targeted effects, affecting various molecular targets including cytokines, transcription factors, growth-factor receptors, adhesion molecules, and inflammatory enzymes. Moreover, the combination of natural compounds with standard chemotherapeutic drugs, namely doxorubicin, cisplatin, and fluorouracil, has considerably improved patient survival by making cancer cells more sensitive to radiotherapy and chemotherapy [3,4,5,6,7,8]. Paclitaxel, vincristine, and etoposide are just a few examples of plant-derived compounds used in therapy. To date, new generations of compounds have been developed and some of these are in clinical use, whereas others are in clinical trials. In recent years, some classes of diterpenes have been investigated for their potential role as anti-cancer agents [9]. In particular, the diterpenoid andrographolide has attracted interest in the medicinal chemistry research community with its potential multiple pharmacological activities such as antioxidant [10,11], anti-inflammatory [12], immuno-regulatory [13], hypoglycemic [14], and antimicrobial [15,16,17,18] properties, and its role in improving memory impairment [19], regulating blood lipids levels, and mitigating cartilage damage [20]. Currently a number of medications and pills containing andrographolide and its derivatives (Figure 1) such as chuanhuning (potassium dehydroandrographolide succinate), yanhuning (potassium sodium dehydroandrographolide succinate), and lianbizhi (andrographolide sodium bisulfite) are available commercially and are utilized in the clinical treatment of diseases such as bacillary dysentery, pediatric pneumonia, and upper respiratory tract infections [21,22,23,24,25].

Considering all the potential benefits of the labdane diterpenoid andrographolide, the aim of the current review was to summarize and describe the most recent studies on the anti-cancer properties of this compound. By analyzing all the studies published within the last ten years, the mechanism of action at the cellular level, pharmacokinetics, and therapeutic potential in cancer treatment, are described. 

## 2. Methodology Adopted for the Current Investigation

Extensive research was conducted into several scientific databases such as PubMed, Scopus, Web of Science, SciFinder, and Science Direct from 2012 to 2022 using the terms “andrographolide”, “*Andrographis*”, “*Andrographis paniculata*”, “cancer,” “signaling pathways”, “apoptosis”, “metastasis”, “synergistic action”, “radiation”, “angiogenesis”, “cell cycle arrest”, “cell cycle regulation”, “cytotoxicity”, “antioxidant”, “breast cancer”, “cervical cancer”, “colorectal cancer”, “toxicity”, and “pharmacokinetics”. 

The inclusion criteria comprised papers that reported the anti-cancer activity of andrographolide, articles published in English, and book chapters that reported studies on cell cultures or animal models with evidence of the mechanisms of action. The exclusion criteria comprised abstracts, case reports, and conference proceedings that did not meet the inclusion criteria. Selected works included data on experimental models, concentration and/or dose, and the analysis of the anti-cancer mechanism of action. 

## 3. Occurrence and Chemistry of the Labdane Diterpenoid Andrographolide

From long ago, the herbaceous plant species *Andrographis paniculata* (Burm.f.) Nees. which belongs to the family Acanthaceae, has been extensively used in the Chinese, Indian, and South-East Asian countries such as Thailand and Vietnam, in the traditional and clinical system of medicine for the treatment of bacterial and viral infections such as cough, sore throat, cold, carbuncle, fever, and sores [21,26]. It has several local names in different countries such as kalmegh in India, boner kalomegh in Bangladesh, Chuan-Xin-Lian in China, hempedu bumi in Malaysia, and fah talai in Thailand [26,27]. It is also known as the known as the “king of bitters”. 

*A. paniculata* has been used for centuries in traditional Asian medicines for the treatment of diarrhea, malaria, flu, leptospirosis, leprosy, rabies, syphilis, upper respiratory infections, sinusitis, HIV infection, and tuberculosis [28,29,30,31]. 

Since 1911, many types of compounds, such as flavonoids, lactones, terpenoids, and diterpenoids, have been identified from different parts of the plant species [32,33]. However, the most common bioactive compound from this plant species is the labdane diterpenoid andrographolides, 14-deoxyandrographolide and 14-Deoxy-11,12-dehydroandrographolide [21,26,34]. As reported by Gorter [32], andrographolide mostly accumulates in the leaves of the plant rather than in other plant parts. Recently, the diterpenoid was also isolated from the leaves of *Andrographis lineata* Wall. ex Nees var.l awii C.B. Clarke [35]. However, *A. paniculata* remains the main source of this promising anti-cancer diterpene lactone.

**Figure 2:**
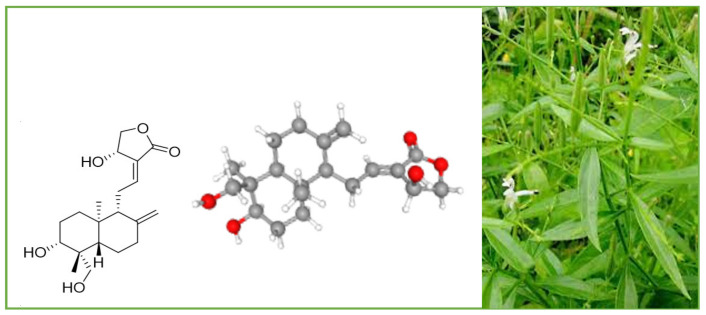
*A. paniculata* and its main compound andrographolide.

Andrographolide (chemical formula: C_20_H_30_O_5_; PubChem CID: 5318517; CAS No. 5508-58-7) is a naturally occurring labdane diterpenoid (Figure 2). It is sparingly soluble in water. 

The other names of andrographolide are (S,E)-4-hydroxy-3-(2-((1R,4aS,5R,6R,8aS)-6-hydroxy-5-(hydroxymethyl)-5,8a-dimethyl-2-methylenedecahydronaphthalen-1-yl)ethylidene)dihydrofuran-2(3H)-one and 3alpha,14,15,18-tetrahydroxy-5b,9bH,10a-labda-8(20),12-dien-16-oic acid gamma-lactone. Its molar mass is 350.455 g/mol and melting point is 230–231 °C.

## 4. Pharmacological Importance of Andrographolide and Its Derivatives

During modern times, andrographolide and several its derivatives have been reported to possess several pharmacological properties, including anti-inflammatory, hepatoprotective, anti-viral, neuroprotective, antioxidant, anti-fibrosis, anti-hyperglycemic, anti-tumor, anti-atherosclerosis, antimicrobial, and cardiovascular protective activities. These properties have been discussed in detail in several outstanding review articles [21,33,36,37,38,39]. Andrographolide exhibits free-radical-scavenging activity and anti-inflammatory effects by inhibition of lipopolysaccharide-induced nitric oxide (NO) production and inducible NO synthase (iNOS) expression, and by suppression of IL-2 production and T-cell proliferation. The lactone diterpene demonstrated its effectiveness in the treatment of Alzheimer’s disease, Parkinsonism, spatial memory deficits, depression, and neuro-inflammation [36]. Jayakumar et al. [37] reported several works that evidenced the promising hepatoprotective effects of andrographolide. The diterpene has been shown to be able to inhibit hepatocyte apoptosis, to attenuate concanavalin-A-induced liver injury, and to protect against ethanol-induced hepatotoxicity in mice with an equivalent efficacy of silymarin. Antiviral activity has been reported against Zika virus, human immunodeficiency virus (HIV), herpes simplex virus (HSV), hepatitis C virus, pestiviruses, and flaviviruses [36,37]. 

## 5. Anti-Cancer Properties and Mechanism of Action of Andrographolide and Its Derivatives

**Figure 3:**
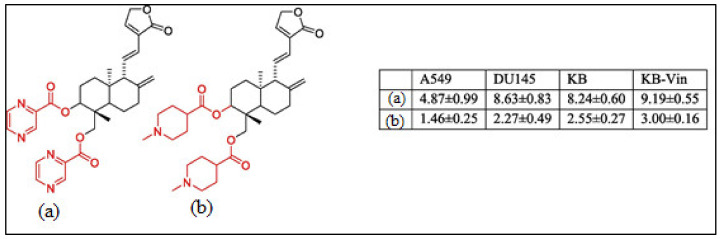
The chemical structure of the characteristic C-3 and C-19 esters of the andrographolide (1) compound with anti-cancer activity; (**a**) & (**b**) are the most active compounds with GI_50_ values of 1.46–9.19 μM against A549 (lung cancer), DU145 (prostate cancer), KB (oral cancer), and KB-Vin tumor cells. Reproduced with permission from Kumar et al. [33], 2020, licensed content publisher—Elsevier.

Recently, much attention has been focused on the anti-tumor/anti-cancer effects of andrographolide and its derivatives and these compounds have been demonstrated to exhibit promising anti-tumor effects in terms of inhibition of the growth, propagation, and relocation of a number of cancerous cells such as prostate carcinoma cells, colon cancer cells, bladder cancer cells, chronic myeloid leukemia cell lines, colorectal cancer cell lines, breast cancer cells, murine leukemia cells, lymphoma, adenocarcinoma PC-3, and leukemic HL-60 cells and many more human cancerous cells (Figure 3) [21,33,40,41,42,43,44,45]. 

**Figure 4:**
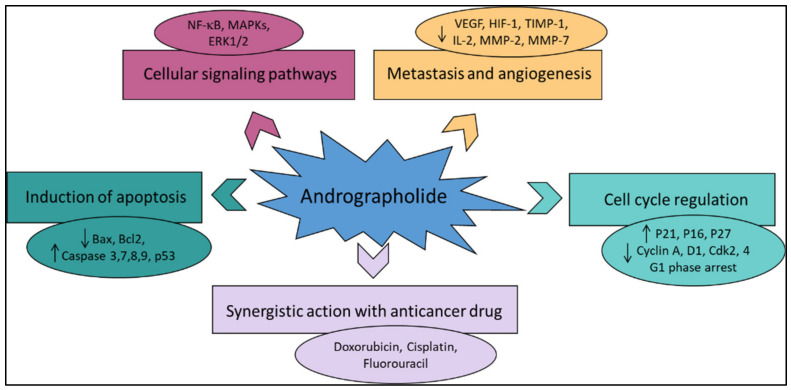
The principal mechanisms of action of andrographolide as an anti-cancer agent.

Various literature reviews have shown that andrographolide and its derivatives was able to reduce cancer cell proliferation/viability and that it is cytotoxic to a broad range of cancer cell lines, but the mechanisms were different for different cell types (Figure 4). Recent published literature has confirmed the multiple anti-cancer effects of the diterpene, in particular against the breast cancer [46], lung cancer [47], colon cancer [48,49], renal carcinoma [50], and cervical cancer [51], as well as hepatoma cancer [52]. The principal mechanism or mode of action of these compounds is less understood and it needs to be explored further in detail. The reduced viability of cancer cells in most of the cases could be partially described by the induction of apoptosis [53,54,55], but in some liver cancer cells the death resulted from the diterpene and was not due to the induction of apoptosis [56].

**Figure 5:**
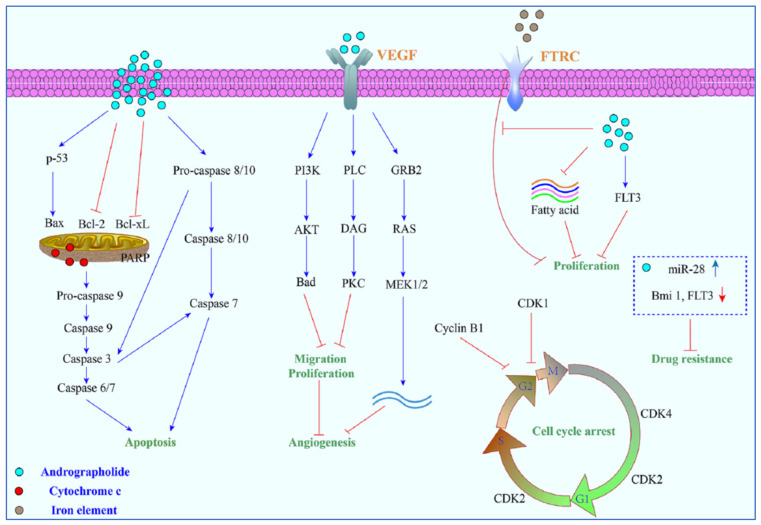
A pictorial representation of the anti-cancer mechanism of action of andrographolide. Reproduced with permission from Zeng et al. [36].

Other mechanisms of action include arrest of the cell cycle and inhibition of cancer angiogenesis. In addition, the enhanced sensitization of cancer cells to radiotherapy is another interesting aspect. Furthermore, the in vivo studies related to the anti-cancer effects of these compounds appear to be only partial and could be further explored. Zeng et al. [36] have also provided a detail pictorial representation of the main anti-cancer mechanisms of action of andrographolide such as the induction of cell apoptosis, blockage of the cell cycle, and inhibition of cancerous cell proliferation (Figure 5). The mechanism of action of the anti-cancer potential of andrographolide and its derivatives, as published during the last ten years (year 2012–2022), are discussed below.

### 5.1. Induction of Apoptosis and Growth Inhibitory Activity

Andrographolide has played a notable role in the recent advancement of pharmacophore development, especially anti-cancer drug development. In anti-cancer-drug development processes, apoptosis induction in carcinoma cells is known to be an important focus [57]. The process of apoptosis in the cell helps in the maintenance of tissue homeostasis by careful exclusion of undesirable cells [58,59]. Andrographolide is credited with potentially inducing apoptosis in several cancer cells and can enhance interleukin-2 secretion by cytotoxic T-lymphocytes for inhibition of tumor growth in mice [60]. 

A recent report on the identified compounds of *A. paniculata* states that andrographolide endorses the apoptosis process in human cancer cells through the induction of mitochondrial cytochrome c, accompanied by enhanced expression of Bax and reduced Bcl-2 in human leukemia HL-60 cells [61] and caspase 8 and caspase 3 activation in human prostate cancer cells PC-3 [62]. It also reported that this compound could inhibit the activity of NF-κB, one of the most important transcription factors accountable for cell proliferation and apoptosis [63,64] (Table 1).

Andrographolide was proposed for potential application in cancer therapy for its apoptosis-induction activity [65]. Andrographolide was found to be accountable for phosphorylation of p53, as well as the transcriptional upregulation of death receptor 4 (DR4) induced by p53. This stimulation process caused the stimulation of the apoptosis via the tumor-necrosis-factor-related apoptosis-inducing ligand (TRAIL). Andrographolide also increased the TRAIL-induced apoptosis process through the DR4 in the TRAIL-resistant cells [65]. Treatment of T-47D mammary cells by andrographolide caused epidermal growth-factor receptor (EGFR) and transferring receptor (TfR) internalization owing to the downregulation of cell-surface receptors and dilapidation of the EGFRs and TfRs [27]. Andrographolide also caused apoptotic cell death by reducing the mRNA and protein levels of IL6 (needed for prostate cancer proliferation) [66]. 

Andrographolide is explicitly studied for its anti-cancer activity and is reported to possess capability to induce cell-cycle arrest in human colorectal carcinoma LoVo cells [67] and to inhibit cell proliferation of cell [68]. 

Induction of apoptosis in human ovarian teratocarcinoma (PA-1) cells was recently described by Bhat et al. [35]. An increased number of cells with activated caspase 3 and a low level of Bcl-2 after treatment with andrographolide was reported in comparison to the untreated cells. Shi et al. [67] described the pharmacophore activity of andrographolide and stated that it arrests the cell at the G1/S phase of the cell cycle via the CKI–cyclin–Cdk network. Andrographolide also showed G0/G1 phase arrest in MCF-7 cells [69]. In addition, 10–30 μM of andrographolide showed pro-apoptotic and growth inhibitory activity in rheumatoid arthritis by G0/G1 phase arrest of the cell cycle via p21 and p27 inhibition, reduced ratio of Bcl2/Bax, and decreased level of CDK-4 protein [70].

The arrest of the G2/M phase of the cell cycle was also caused by the action of andrographolide in glioblastoma U251 and U87 [71], human leukemia (K562) [17] and breast cancer cells [72,73]. Similarly, 3,19-(3-chloro-4fluorobenzylidene) and 3,19-(2-bromobenzylidene), derivatives of andrographolide exhibited superior cytotoxic and growth-inhibition activity in HCT- 116 and MCF-7 cell lines. Both derivatives showed potent inhibitory activity by arrest in the G1/S phase of the cell cycle and apoptosis in MCF-7 and HCT-116 cells [74]. 

Recent research has shown that andrographolide inhibits cell-cycle progression at the G2/M checkpoint in LNCaP, C4-2b, and PC3 cells and at the G1/S checkpoint in DU-145 cells. Cyclin B1 was also upregulated by andrographolide in LNCaP and PC3 cells [75].

Wang et al. [76] have shown that the osteosarcoma cell proliferation was inhibited by andrographolide by the process of arresting of the cell cycle at the G2/M phase and by enhancing the caspase-mediated apoptosis process. In vitro andrographolide inhibited the growth of osteosarcoma cells by causing G2/M phase cell-cycle arrest and inducing apoptosis via the reactive oxygen species (ROS)/c-Jun N-terminal kinase (JNK) signaling pathway.

In vivo, andrographolide exhibited significant anti-tumor activity with minimal toxicity. 

### 5.2. Inhibition of Tumor Angiogenesis

The term tumor angiogenesis refers to the formation of new blood vessels within a tumor, which provide the growth center with a constant supply of oxygen and nutrients. Andrographolide decreased tumor-specific angiogenesis by lowering the manufacture of the pro- and anti-angiogenic factors such as interleukin-2, vascular endothelial growth factor, nitric oxide, and tumor necrosis factor TNF-α, in the C57BL/6 mice infected with the B16F-10 melanoma cells (Table 1) [75]. In addition, it was able of inhibit the angiogenesis-critical matrix metalloproteinase 2 (MMP-2) and metalloproteinase 9 (MMP-9) activities in colon cancer cells [77]. When it comes to A549 cells and non-small-cell lung cancer (NSCLC), HIF-1 is responsible for cancer growth. 

Andrographolide inhibited HIF-1, reduced vascular endothelial growth factor (VEGF), and boosted hydroxyl-HIF-1 and prolyl hydroxylase expression [78]. These results emphasize the promise of andrographolide as a potential chemotherapeutic or anti-angiogenesis drug for the treatment of NSCLC. Serum levels of tissue inhibitors of metalloproteinase 1 (TIMP-1), VEGF, and pro-inflammatory cytokines such as TNF-α, IL-1b, and IL-6, and granulocyte monocyte colony stimulating factor (GM-CSF) were decreased by andrographolide after being induced by the B16F-10 melanoma cell line in the C57BL/6 mice [60]. 

The andrographolide derivative 17-hydro-9-dehydro-andrographolide inhibited vascular endothelial cell proliferation and angiogenesis in rats at 1–10 mM [79]. At 50 mg/kg, andrographolide inhibited the expression of PCNA, vascular endothelial growth factor, and cyclin D1 in hamster buccal cells [38]. In another study, it is stated that biochemical analysis had identified andrographolide as a significant docking molecule that can bind to the ATP-binding pocket of vascular endothelial growth-factor receptor (VEGFR2) and thus inhibit its kinase activity by potentially interacting with the kinase domain of VEGFR2 [80]. VEGFR2 is the major receptor of VEGF. It is expressed in vascular endothelial cells and plays a very significant role in angiogenesis. In fact, by binding and activating VEGFR2, VEGF mediates endothelial invasion, migration, cell proliferation, and survival, and increases vascular permeability and neo-vascularization. In addition, in another study the author discussed the inhibition of angiogenesis by andrographolide by the process of inhibition of the Mir-21-5p/TIMP3 signaling pathway [81]. The results showed that andrographolide was able to inhibit the growth of the vascular tissues in the membranes of chick embryo chorioallantois and yolk sac, along with the suppression of the tumor angiogenesis [81]. Furthermore, they also stated that the proliferation, migration, and tube formation of the vascular endothelial cells was also inhibited by andrographolide action under in vitro action. The outcome of the process was principally facilitated through the inhibition of the expression of miR-21-5p and added targeting of the TIMP3; this proved that andrographolide was directly involved in the inhibition of angiogenesis [81]. Another article discussed the overall mechanism of action of andrographolide by the inhibition of the PI3K/AKT, NF-κB, v-Src, and STAT3 activities followed by the downregulation of the mediators of progression of the cell cycle, metastasis, and angiogenesis [82]. 

**Figure 6:**
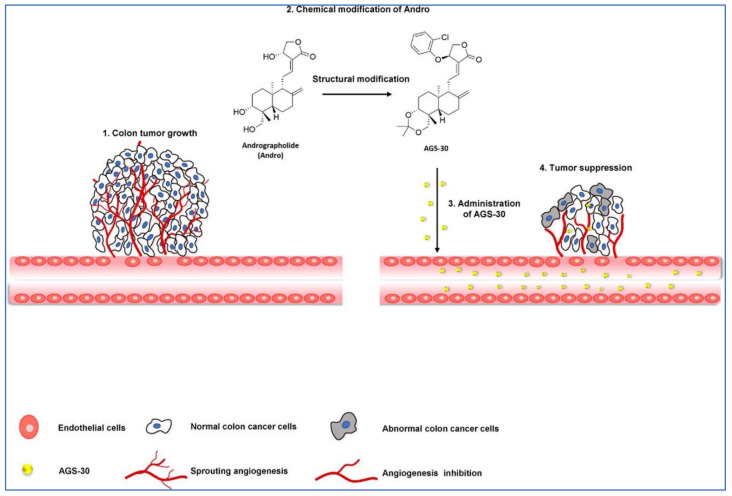
Pictorial representation of action mechanism of AGS-30, an andrographolide derivative, suppressing the tumor angiogenesis process. Reproduced with permission from Li et al. [83].

In a study by Li et al. [83], the author stated that a new andrographolide derivative (AGS-30) was able to display anti-angiogenic properties through the inhibition of the endothelial-cell proliferation, incursion, and relocation, as well as tube formation (Figure 6).

The author also stated that the AGS-30 was able to inhibit cell proliferation and the phosphorylation of cell-survival-related proteins followed by the reduction of the VEGF expression in the HT-29 colon cancer cells [83]. Moreover, AGS-30 also suppressed the tumor growth and angiogenesis process in the HT-29 colon cancer cell xenografts in nude mice [83].

### 5.3. Anti-Proliferative Activity

The anti-proliferative effects of andrographolide have been investigated against several cancer cell lines (Table 1). Udomwan et al. [84] investigated the cytotoxic activity of andrographolide by using the 3-(4,5-dimethylthiazole-2-yl)-2,5-biphenyl tetrazolium bromide (MTT) assay against three cervical cancer cell lines, namely CaSki, SiHa, and C33A. The most sensitive cells after treatment with andrographolide (at 20, 40, 80, and 160 µM concentrations) for 48 h were SiHa cells. Cell viability of SiHa cells was reduced to 50% at a concentration of 85.59 µM followed by the value of 87.52 µM for CaSki cells, and 96.05 µM for C33A cells. 

Successively, Tohkayomatee et al. [85] assessed the andrographolide effects on the cell viability against MCF-7 and MDAMB-231 breast cancer cell lines by MTT assay. At concentrations in the range 7.5–120 µM, the diterpene considerably reduced, in a concentration- and time-dependent manner, the cell viability of both cell lines with IC_50_ values after 48 h of exposure of 32.90 and 37.56 µM against MCF-7 and MDAMB-231, respectively. 

Treatment with andrographolide at the concentration of 50 µM reduced the viability of the THP-1 (human monocytic leukemia) cell line and NCI-H929 (human IgAkappa-producing multiple myeloma) cell line to 39.2 and 13.0%, respectively, with respect to the untreated cells in a concentration-dependent manner [86]. The IC_50_ values for treating were 31 and 8 µM, for THP-1 and H929 cells, respectively. Andrographolide was demonstrated to be active also against human malignant melanoma A375 and C8161 cell lines [87]. The cell viability was assessed by MTT assay. The IC_50_ values after 48 h of exposure were 12.07 and 10.92 µM for A375 and C8161, respectively, suggesting the promising anti-proliferative activity of andrographolide against malignant melanoma cells in a concentration- and time-dependent manner. 

Previously, the anti-proliferative activity of andrographolide on HT-29 colon cancer cells was studied by using MTT assay, colony formation assay, trypan blue exclusion assay, and morphological analysis [55]. The diterpene reduced cell viability of HT-29 cells in a concentration- and time-dependent manner. An interesting IC_50_ value of 3.7 µg/mL was found against human ovarian teratocarcinoma (PA-1) cells [35]. 

Devendra et al. [43], demonstrated that a series of 3,19-O-acetal derivatives of andrographolide exhibited significant anti-cancer properties and the results specified that the protection of the 3,19-hydroxyl groups of andrographolide with the appropriate ethylidene/benzylidene moiety prompted a substantial cytotoxicity effect with either the acetylation or dehydration of the 14-hydroxyl of the lead compound cyclic acetal derivative, that could have triggered its cytotoxic effect on all the cell lines [43]. 

### 5.4. Induction of Autophagy

Autophagy is a process associated with several diseases, including the cancer that destroys and processes the damaged macromolecules and organelles through lysosomal pathways to maintain the homeostasis of cells [88]. Autophagy may play contrasting roles in different types of cancers and in their different stages of development, including promoting survival and inducing death [89]. For this reason, research into the molecular mechanisms of autophagy-related signal-transduction pathways is of interest [90,91,92,93]. In this context, Liu et al. [94] demonstrated the effects on autophagy of andrographolide in human osteosarcoma cells by suppressing the phosphatidylinositol-3-kinase (PI3K)/Akt and the mammalian target of rapamycin (mTOR) signaling pathways and enhancing the c-Jun N-terminal kinase (JNK) pathway. Autophagy induced by andrographolide inhibits the invasion and metastasis of osteosarcoma cells suggesting that the diterpene may represent a promising targeted agent in the prevention and treatment of osteosarcoma. Yuwen et al. [42] demonstrated that cisplatin induced autophagy that attenuated the sensitivity of both A549 and Lewis lung cancer cells to cisplatin, whereas the clinical drug andrographolide suppressed autophagy and enhanced cisplatin-mediated apoptosis in these cells [42].

### 5.5. Oxidative Stress and Antioxidant Properties of Andrographolide in Anti-Cancer Treatment 

Andrographolide exhibited promising antioxidant properties acting through different mechanisms of action including the neutralization of free radicals, the activation of antioxidant enzymes, the inhibition of pro-oxidant enzymes, and the protection of mitochondrial integrity [95]. Some studies have shown the reduction of ROS in cellular models by andrographolide [96,97]. Treatment with andrographolide (10 and 30 M) decreased the production of ROS in RAW264.7 macrophages motivated by the ovalbumin or lipopolysaccharide (LPS) [95]. Previously, andrographolide has been reported to inhibit intracellular ROS production in *N*-formylmethionyl-leucyl-phenylalanine-induced neutrophils [96]. Zhan et al. [98] showed a dose-dependent increase in catalase (CAT) and super oxide dismutase (SOD) activities after topical application of andrographolide to mouse skin that was exposed to UV radiation, as compared to the untreated mice.

Andrographolide is also considered to be a potent inhibitor of the enzyme xanthine oxidase (XOD), that catalyzes the terminal steps of purine degradation. XOD has been proposed as a source of oxygen radicals in epithelial, connective, and endothelial tissue cells. In fact, the enzyme is described as playing an important role in cellular oxidative status. An in silico study revealed strong binding interactions between the andrographolide and the XOD enzyme [99] and a recent work showed the ability of the diterpene (30 mg/kg/day) to reduce liver XOD activity [100].

NADPH oxidase (NOX) is a membrane enzyme complex that catalyzes the oxidation reaction of NADPH by oxygen, inducing ROS production in cells. Andrographolide has been reported to reduce the expression of NOX2 through limiting the activation of phosphoinositide 3-kinase/protein kinase B (PI3K/AKT)-dependent nuclear factor-kappa B (NF-B) [101]. In another work, andrographolide (10 and 20 mg/kg/day) significantly decreased NOX2 and NOX4 expression in myocardial tissues [102]. There are reports that andrographolide can improve mitochondrial dysfunction in some in vitro and in vivo models. Geng et al. [103] demonstrated that andrographolide sulfonate treatment could reduce oxidative stress and protect the mitochondria in a transgenic mouse model (amyloid precursor protein/presenilin 1). In another model, it was found that andrographolide supplementation could reduce the production of nitric oxide, carbonyl protein, and malondialdehyde, and enhance mitochondrial complex activities in the electron transport chain [104]. On the other hand, increased activity of CAT, SOD, glutathione peroxidase, glutathione reductase, and glutathione-*S*-transferase, and reduced concentrations of glutathione and glutathione disulfite were observed [104]. 

**Table 1:** In vitro and in vivo study of andrographolide against cancer cell lines.

Cancer Cell Line	Study Type	Main Effects	Ref.
Ovarian teratocarcinoma	In vitro	PA-1 cells—MTT test—IC_50_ 3.7 µg/mL; induction of apoptosis	[35]
Lung cancer	In vitro	Suppression of autophagy and enhanced cisplatin-mediated apoptosis	[42]
Colon cancer	In vitro	HT-29 cells—induction of programed cell death and cell-cycle arrest through the increase of intracellular ROS level	[55]
Leukemia	In vitro	HL-60 cells—cell-cycle arrest and mitochondrial-mediated apoptosis	[61]
Prostate cancer	In vitro	PC-3 cells—caspase 8 and caspase 3 activation	[62]
Neuroblastoma	In vitro	Induction of p53- and caspase-independent cell death	[63]
Liver cancer	In vitro	HepG2 and Hep3B cells—sensitization of cancer cells to TRAIL-induced apoptosis via p53-mediated death receptor 4 upregulation	[65]
Cervical cancer	In vitro	HeLa cells—sensitization of cancer cells to TRAIL-induced apoptosis via p53-mediated death receptor 4 upregulation	[65]
Colorectal cancer	In vitro	HCT116 cells—sensitization of cancer cells to TRAIL-induced apoptosis via p53-mediated death receptor 4 upregulation	[65]
Prostate cancer	In vitro	LNCaP, DU145, and PC-3 cells—apoptotic cell death by reducing the mRNA and protein levels of IL6	[66]
Colorectal carcinoma	In vitro	LoVo cells—induction of cell-cycle arrest and inhibition of cell proliferation	[67,68]
Brest cancer	In vitro	MCF-7 cells—G0/G1 phase arrest	[69]
Glioblastoma		U251 and U87 cells—arrest of the G2/M phase cell cycle phase	[71]
Leukemia		K562 cells—arrest of the G2/M cell cycle phase	[17]
Breast cancer		Arrest of the G2/M phase	[72,73]
Prostate cancer	In vitro	LNCaP, C4-2b, and PC3 cells—inhibition of cell-cycle progression at the G2/M phase; decrease of tumor-specific angiogenesis	[75]
Osteosarcoma	In vitro	Inhibition of cell proliferation by arresting of the cell cycle at the G2/M phase	[76]
Osteosarcoma	In vivo	Female Balb/c-nu mice with HOS-Luc cells injected subcutaneously—induction of apoptosis via the ROS/JNK pathway	[76]
Colon cancer	In vitro	HT29 cell line—anti-invasive activity against colon cancer cells via inhibition of matrix metalloproteinase 2 (MMP2)	[77]
Cervical cancer	In vitro	CaSki cell line—MTT test—reduction of cell viability of 50% at 87.52 µM	[84]
Cervical cancer	In vitro	SiHa cell line—MTT test—reduction of cell viability of 50% at 85.59 µM	[84]
Cervical cancer	In vitro	C33A cell line—MTT test—reduction of cell viability of 50% at 96.05 µM	[84]
Breast cancer	In vitro	MCF-7 cell line—MTT test—IC_50_ 32.90 µM	[85]
Breast cancer	In vitro	MDAMB-231 cell line—MTT test—IC_50_ 37.56 µM	[85]
Monocytic leukemia	In vitro	THP-1 cell line—MTT test—IC_50_ 31 µM	[86]
IgAkappa-producing multiple myeloma	In vitro	H929 cell line—MTT test—IC_50_ 8 µM	[86]
Malignant melanoma	In vitro	A375 cell line—MTT test—IC_50_ 12.07 µM	[87]
Malignant melanoma	In vitro	C8161 cell line—MTT test—IC_50_ 10.92 µM	[87]
Osteosarcoma	In vitro	Suppression of phosphatidylinositol-3-kinase (PI3K)/Akt and the mammalian target of rapamycin (mTOR) signaling pathways; enhancement of the c-Jun N-terminal kinase (JNK) pathway	[94]
Mice	In vivo	Inhibition of PI3K/AKT-dependent NOX2 and iNOS expression	[101]
APP/PS1 transgenic mice	In vivo	Reduction of oxidative stress and protection of mitochondria	[103]
Male Wistar rats	In vivo	Reduction of the production of nitric oxide, carbonyl protein, and malondialdehyde, and enhancement of mitochondrial complex activities in the electron transport chain. Increased activity of CAT, SOD, glutathione peroxidase, glutathione reductase, and glutathione-S-transferase	[104]

The transcription factor NF-E2-related factor 2 (Nrf2), takes part in regulation of the antioxidant defense system. Thus, the involvement of andrographolide in the regulation of Nrf2 is a topic of interest in redox-system regulation. Wong et al. [105] investigated the mechanism through which andrographolide is able to activate the transcription of Nrf2, finding that this compound was able to inhibit the Keap1 protein which along with the Cul3 and RBX1 forms an E3 ubiquitin ligase that polyubiquitinates Nrf2. In particular, andrographolide partly inhibits Keap1 and Cul3 interactions. Fu et al. [106] confirmed that andrographolide activates the Nrf2 signaling pathway. The author also studied the mRNA expression of NQO-1 and HO-1, which are the target genes of Nrf2, establishing that their mRNA expression levels were significantly increased.

Andrographolide and its derivative compounds are found to exhibit reactive oxygen species (ROS)-mediated apoptotic cell death in a number of cell lines, including mantle cell lymphoma, diffuse large B-cell lymphoma cell line SUDHL4, Burkitt p53-mutated Ramos, follicular lymphoma HF-1, and primary cells acquired from the patients with such diseases [38,41]. Further, another research by Chen et al. [107], showed that carboxylic-acid-mediated cytotoxic activity against the HCT-116 and MCF-7 cell lines was exerted by the andro-19-oic acid derivatives [108]. Andrographolide–lipoic acid conjugate was also reported to display anti-cancer cytotoxicity effects against the human leukemia K562 cells through the excess production of ROS followed by DNA damage and mitochondria-facilitated apoptosis [109]. Banerjee et al. reported on the cytotoxic effects of andrographolide against breast cancer cells (MDA-MB-231 and MCF-10A) by upregulation of Apaf-1 and Bax proteins and downregulation of Bcl-xL and Bcl-2 proteins that resulted in an increase in ROS activity [53]. Similarly, ROS-mediated cytotoxic potential by the andrographolide sodium bisulfate in human renal tubular epithelial cells was also reported. This activated the c-Jun N-terminal kinase signaling pathway [110].

### 5.6. Enhanced Radio-Sensitivity

Different in vitro and in vivo studies have suggested that andrographolide was also able to enhance the sensitization of cancer cells to radiotherapy [107,111,112,113,114]. Zhang et al. [115] examined the radio-sensitizing activity of andrographolide in human ovarian SKOV3 xenografts analyzing the effects of the diterpene administration on apoptosis, cancer growth, autophagy, and radiosensitivity. Andrographolide strongly sensitized ovarian SKOV3 xenografts to radiation. Moreover, the authors demonstrated that autophagy and apoptosis in radiation, combined with drug treatment, was considerably increased compared with radiation treatment or drug administration alone. An increase in the p-p53 expression and the Bax/Bcl-2 protein ratio after the combination radiation–andrographolide treatment was observed. The radio-sensitizing activity of andrographolide on ECA109 esophageal cancer cells was also found by using the clonogenic survival assay [111]. Andrographolide could markedly enhance radio-sensitivity with a sensitizing enhancement ratio of 1.28. This effect may be associated with the induced apoptosis of ECA109 cells and the decrease in the levels of nuclear factor kappa B (NF κb). 

Previously, Hung et al. [112] measured the radio-sensitizing effects of andrographolide in H-ras-transformed rat kidney epithelial (RK3E) cells. Ras is one of the well-characterized proto-oncogenes that control multiple intracellular signaling networks including NF- κb, mitogen-activated protein (MAP) kinase, and phosphoinositide-3-kinase (PI3K)/protein kinase B (Akt) pathways. Ras-regulated signal pathways control proliferation, differentiation, apoptosis, actin cytoskeletal integrity, cell adhesion, and cell migration. Moreover, Ras activation has been shown to increase the radio-resistance of cancer cells. Andrographolide sensitized Ras-transformed cells to radiation in both in vitro and in vivo models. This radio-sensitization was associated with downregulation of Akt and NF-kB activity. Andrographolide combined with radiation exhibited synergistic effects, suppressing tumorigenesis in oral cancer stem cells (OCSCs) and cells characterized by high tumorigenic and metastatic properties as well as chemo-resistance and radio-resistance [113]. More recently, the diterpene plus radiation increased apoptosis and decreased survival and invasion of HCT116 colorectal cancer cells compared with the effects of radiation alone [114].

### 5.7. Different Signalling Pathways

Different signaling pathways in which andrographolide inhibited tumor growth are discussed in a few more studies. In one study, the author reported the effects of andrographolide on TNF-α-induced IL-8 expression and its principal mode of action. The author concluded that andrographolide inhibited TNF-α-induced IL-8 mRNA, and reduced IL-8 transcriptional activity and protein expression in a concentration dependent manner [116].

**Figure 7:**
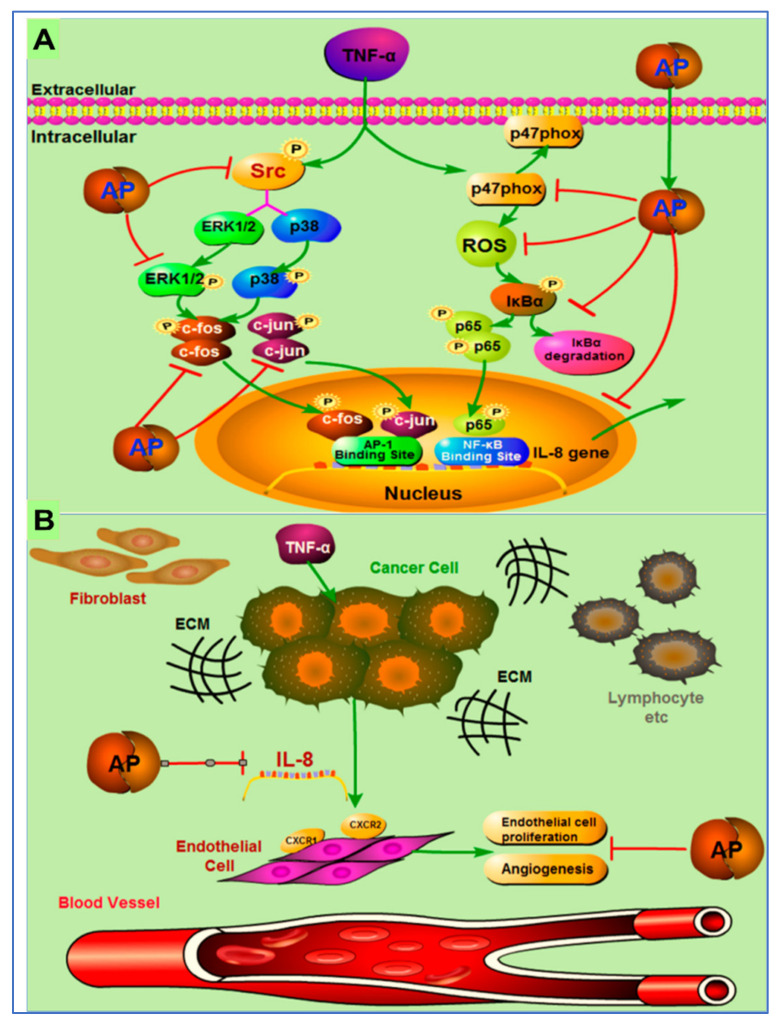
Pictorial representation of mechanism of action of inhibition of tumor-derived IL-8-induced angiogenesis by andrographolide in the tumor microenvironment. (**A**) Andrographolide mediates inhibition of TNF-α-induced IL-8 through the inhibition of NADPH oxidase/ROS/NF-κB and Src/MAPKs/AP-1signaling pathways in the HCT116 colorectal cancer cells. (**B**) Secretion of the IL-8 from cancer cells increases the multiplication of endothelial cells to endorse the angiogenesis process in the tumor microenvironment. Andrographolide obstructs the countenance of tumor-derived IL-8, thus preventing angiogenesis in the tumor microenvironment. Reproduced with permission from Yuan et al. [116]. Copyright © 2023, American Chemical Society.

The possible mechanism of action of andrographolides is presented in Figure 7. They demonstrated that andrographolide efficiently suppressed IL-8 expression and angiogenesis in the tumor microenvironment by inhibition of NFκB, Erk1/2, NADPH oxidase, ROS, and P38 MAPK, and activation of AP-1 [116].

**Figure 8:**
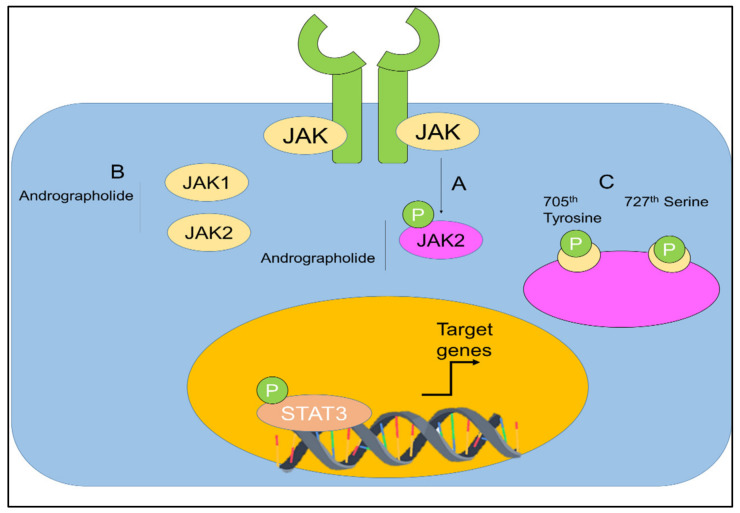
(**A**,**B**) Regulation of JAK-STAT signaling by andrographolide. Andrographolide effectively inhibited JAK1, JAK2, and STAT3. (**C**) Andrographolide inhibited phosphorylation of STAT3 on the 705th tyrosine and 727th serine. Adopted from Farooqi et al. [120], under the terms and conditions of the Creative Commons Attribution (CC BY) license (http://creativecommons.org/licenses/by/4.0/), 2020. Licensee MDPI, Basel, Switzerland.

The capability of andrographolide to control the signal transducer and activator of transcription (STAT) proteins in cancers was also investigated (Figure 8) [67,117,118,119].

Chun et al. [67], stated that, in a dose-dependent manner, andrographolide was able to inhibit the interleukin-6 (IL-6) at mRNA and protein levels. Andrographolide was able to suppress the IL-6 autocrine- and paracrine-loop-mediated signaling pathways primarily through the process of disturbing the phosphorylation of STAT3 and extracellular signal regulated kinase. It was shown that andrographolide encouraged apoptosis in the androgen-stimulated and castration-resistant prostate cancer cells [67]. In addition, andrographolide was reported to have inhibited the tyrosine phosphorylation of JAK1 and JAK2 [118]. Andrographolide was also found to be effective against pancreatic cancer cells [119]. 

## 6. Pharmacokinetics Properties

Currently, studies related to andrographolide are still in their primary stages and there are a number of challenges in the optimization of therapeutic applications and the bioavailability of this compound. Its poor pharmacokinetic effects, such as the metabolism, fast absorption, and elimination, have resulted in its low availability [21]. The low bioavailability of andrographolide, has led to the development of its derivatives and other modern techniques such as nanotechnology and nanoencapsulation [115,120,121].

Many factors, including pharmacokinetics, can influence the efficacy of a drug. Pharmacokinetics relates to the passage of drugs through the body (absorption, distribution, and elimination). Several studies have been carried out on the pharmacokinetic parameters of andrographolide. After oral administration of andrographolide at 100 mg/kg/day, for 4 weeks in Wistar rats, Bera et al. [122] identified the highest concentration of andrographolide was in kidney (156.12 ng/g) followed by the spleen, liver, and brain while almost the same concentration was found in the heart and lungs. An apparent C_max_ value of 115.81 ng/mL and an elimination half-life (t1/2) of 0.75 h were found. Reduction of the concentration of orally administered andrographolide to 60 mg/kg/day in the same model caused a reduction in the apparent C_max_ value to11.52 µg/mL and a T_max_ of 2.01 with a clearance value of 0.19 L/h/kg [123]. 

The low oral bioavailability is probably caused by the rapid metabolism to 14-deoxy-12-suloandrographolide in the duodenum and jejunum and probably in the last part of the ileum or colon. Another factor that negatively influenced andrographolide bioavailability is the efflux of P-glycoproteins [124]. The same research group also tested andrographolide at the same dosage in Sprague Dawley (SD) rats and found an apparent C_max_ value of 9.73 µg/mL and a t1/2 of 7.30 with a clearance value of 0.03 L/h/Kg and an AUC0-t of 67.19 h/μg/mL [125]. Intravenous injection in SD of andrographolide at dosage of 80 mg/kg resulted in an elimination half-life (t1/2) of 0.4 h with a clearance value of 0.7 L/h/kg [126].

Using a beagle dog model, andrographolide administered by intravenous injection at the dosage of 50 mg/kg showed a t1/2 of 0.828 h with a clearance value of 1.02 L/h/kg [127]. Regarding the distribution pattern, Godugu et al. [128] demonstrated that andrographolide interacted with human serum albumin amino acid residues by forming hydrogen bonds with Agr218, Trp214, and Lys444. Moreover, Zhao et al. [129] identified eight phase I and five phase II metabolites resulting from dehydration, deoxygenation, hydrogenation, and glucuronidation reactions. 

Recently, Yu et al. [130] clarified that the α-β-unsaturated lactone moiety was metabolized mainly by CYP3A4 whereas conjugation reactions were mediated by uridine diphosphate glucuronyltransferase (UGT) (UGT1A3, UGT1A4, UGT2B4, and UGT2B7). Preclinical pharmacokinetic studies revealed that andrographolide was excreted via the urine only at a lower amount of 7–9%, and the remaining aliquot was eliminated through different routes [131]. The following creatinine adducts were identified in human urine: 14-deoxy-12-(creatinine-5-yl)-andrographolide-19-*O*-βd-glucuronide A and 14-deoxy-12-(creatinine-5-yl)-andrographolide-19-*O*-β-d-glucuronide B [132]. Several studies have proved the interaction between andrographolide and other drugs. For instance, Zhang et al. [133] investigated the effect of andrographolide and warfarin co-administration and found an increase in the systemic exposure of warfarin in rats from 60.58 to 118.92 µg h/mL and a t1/2 from 14.27 to 22.73 h. The authors demonstrated the ability of andrographolide to inhibit CYP3A4 and CYP2C9, which are responsible for warfarin metabolism. Another important interaction was observed in a hepatocellular carcinoma (HepG2) model in which andrographolide reduced the expression of CYP2D6 and influenced the pharmacokinetic parameters of 5-fluorouracile [134]. 

Clinical pharmacokinetics data are consistent with findings derived from animal studies. For example, a study carried out by Pholphana et al. [135] in volunteers who received an equivalent dose of 97.92 mg/day of andrographolide for three consecutive days, showed a T_max_ value of 0.78 h. This study has also showed that the metabolites of andrographolide 14-deoxy-11, 12-didehydroandrographolide had an AUC value and a C_max_ greater than that of andrographolide. These results could suggest that the metabolites of andrographolide contribute to the biological activity of this diterpene. Moreover, data on apparent clearance demonstrated that there are no significant differences between males and females.

## 7. Adverse Effects

One of the main adverse effects registered for andrographolide, independently of the route of administration, was the nephrotoxicity. In fact, andrographolide was able to inhibit human renal tubular epithelial (HK-2) cell proliferation and to induce apoptosis, as well as increase the content of malondialdehyde (MDA) and decrease the expression of SOD. Moreover, andrographolide increased C/EBP homologous protein (CHOP) and caspase-4 by inducing damage at the endoplasmic reticulum level [136]. Successively, Liang et al. [137] have demonstrated that andrographolide reduced the reproductive capacity of female rodents and caused the apoptosis of most oocytes. In addition, mild adverse events, including rash and taste disturbance, emerged from a clinical study in which andrographolide was orally administered twice daily at a dose of 140 mg [138]. 

Calabrese et al. [139] demonstrated that andrographolide administered orally for 3 weeks at a dose of 5 mg/kg body weight, escalating to 10 mg/kg bodyweight for 3 weeks, and to 20 mg/kg bodyweight for a final 3 weeks caused mild to moderate headache, soreness, rash, taste symptoms, diarrhea, or itching anaphylactic reaction. The acute and subacute toxicity of AG-2-HyP-β-CYD complex on SD rats following oral and inhalation routes of administration was recently tested [140]. The lethal dose (LD_50_) was found to be > 2000 mg/kg in addition to NOAEL (no observed adverse effect level) of 666 mg/kg. In general, knowledge of the toxicity of andrographolide is still rather limited as studies have focused only on the single molecule and not on its metabolites or on its co-administration of other drugs. However, the major interest in this molecule is linked to its promising antitumor activity [61,141,142]. 

## 8. Clinical Trial on Andrographolide

A number of clinical research investigations and trials on the application of andrographolide and its derivatives in the treatment of several diseases such as multiple sclerosis, tonsillitis, bronchitis, migraine, COVID-19, osteoarthritis, arthritis, rheumatoid, and cancers are on-going (https://clinicaltrials.gov/ct2/results?cond=&term=Andrographolide&cntry=&state=&city=&dist=, accessed on 4 May 2023). However, we found that only 20 clinical trials on andrographolides have been reported (https://clinicaltrials.gov/ct2/results?cond=&term=Andrographolide&cntry=&state=&city=&dist=, accessed on 4 May 2023), and among them, only two were cancer-related clinical research. In one clinical trial the authors studied the effectiveness and protection effects of andrographolides mixed with capecitabine in the treatment of elderly patients with locally progressive, recurring, or metastasis-inoperable colorectal cancers. However, the trial was terminated after the phase 2 trials due to the low accuracy rate of the results (ClinicalTrials.gov Identifier: NCT01993472), (https://clinicaltrials.gov/ct2/show/NCT01993472?term=Andrographolide&draw=3&rank=2, accessed on 4 May 2023). In another study, the authors investigated the effect of *Andrographis paniculata* on the palliative organization of patients with progressive or metastatic esophageal cancer. In this study, around 30 patients with locally advanced or metastatic squamous esophageal cancer were taken into consideration and the study was completed after a successful phase 3 trial (ClinicalTrials.gov Identifier: NCT04196075) (https://clinicaltrials.gov/ct2/show/NCT04196075?term=Andrographolide&draw=3&rank=6, accessed on 4 May 2023). 

## 9. Conclusions and Future Perspectives

Among the numerous natural compounds investigated as potential anti-cancer drugs, the diterpene lactone andrographolide has shown promising activity through different mechanisms of action, mainly including induction of apoptosis. Moreover, some studies have examined the combination of andrographolide with other chemotherapeutic drugs and radiation in the treatment of malignant cancers. 

Currently, some projects related to clinical research on andrographolide for the treatment of colorectal cancer and esophageal carcinoma, as well as the evaluation of its potential role in the treatment of primary progressive multiple sclerosis, acute tonsillitis, and bronchitis, are in progress. In addition, several research studies have proposed andrographolide derivatives characterized by a reduced toxicity and increased therapeutic efficacy. However, it is necessary to carry out large-scale trials to verify the exact efficacy of andrographolide and its pharmacokinetic parameters, and this could be an obstacle in the clinical product transformation. Indeed, one of the challenges in the field of the bioactivity of andrographolide is that it exhibits low bioavailability and poor solubility. Extensive chemical modifications have been carried out in order to develop andrographolide derivatives that have improved bioavailability and solubility. Moreover, in recent years, mesoporous silicon, liposomes, and nanoparticles have represented a strategy to ameliorate the pharmacokinetics of andrographolide. 

Overall, a number of studies and some clinical trials on andrographolide could bring a robust sureness to the use andrographolide in the inhibition and cure of associated diseases, and this will endorse and hasten clinical studies of andrographolide and its derivatives in the formulation of modern drugs. 

## References

[1] Hassanpour S.H., Dehghani M. (2017). Review of cancer from perspective of molecular. J. Cancer Res. Pract..

[2] World Health Organization (WHO) Latest Global Cancer Data: Cancer Burden Rises to 19.3 Million New Cases and 10.0 Million Cancer Deaths in 2020. https://www.iarc.who.int/news-events/latest-global-cancer-data-cancer-burden-rises-to-19-3-million-new-cases-and-10-0-million-cancer-deaths-in-2020/.

[3] Huang M., Lu J.J., Ding J. (2021). Natural products in cancer therapy: Past, present and future. Nat. Prod. Bioprospect..

[4] Cragg G.M., Grothaus P.G., Newman D.J. (2009). Impact of natural products on developing new anti-cancer agents. Chem. Rev..

[5] Saeed M.E.M., Boulos J.C., Elhaboub G., Rigano D., Saab A., Loizzo M.R., Hassan L.E.A., Sugimoto Y., Piacente S., Tundis R. (2019). Cytotoxicity of cucurbitacin E from *Citrullus colocynthis* against multidrug-resistant cancer cells. Phytomedicine.

[6] Tundis R., Bonesi M., Deguin B., Loizzo M.R., Menichini F., Conforti F., Tillequin F., Menichini F. (2009). Cytotoxic activity and inhibitory effect on Nitric Oxide production of triterpene saponins from the roots of *Physospermum verticillatum* (Waldst & Kit) (Apiaceae). Bioorg. Med. Chem..

[7] Loizzo M.R., Tundis R., Statti G.A., Menichini F. (2007). Jacaranone: A cytotoxic constituent from *Senecio ambiguus* subsp. *ambiguus* (Biv.) DC. against renal adenocarcinoma ACHN and prostate carcinoma LNCaP cells. Arch. Pharm. Res..

[8] Nobili S., Lippi D., Witort E., Donnini M., Bausi L., Mini E., Capaccioli S. (2009). Natural compounds for cancer treatment and prevention. Pharmacol. Res..

[9] Acquaviva R., Malfa G.A., Loizzo M.R., Xiao J., Bianchi S., Tundis R. (2022). Advances on natural abietane, labdane and clerodane diterpenes as anti-cancer agents: Sources and mechanisms of action. Molecules.

[10] Mussard E., Cesaro A., Lespessailles E., Legrain B., Berteina-Raboin S., Toumi H. (2019). Andrographolide, a natural antioxidant: An update. Antioxidants.

[11] Zhang X.F., Ding M.J., Cheng C., Zhang Y., Xiang S.Y., Lu J., Liu Z.B. (2020). Andrographolide attenuates oxidative stress injury in cigarette smoke extract exposed macrophages through inhibiting SIRT1/ERK signaling. Int. Immunopharmacol..

[12] Ding Y., Chen L., Wu W., Yang J., Yang Z., Liu S. (2017). Andrographolide inhibits influenza A virus-induced inflammation in a murine model through NF-kappaB and JAK-STAT signalling pathway. Microbes Infect..

[13] Zhang B., Yang B., Du L., Guo Y. (2020). Nitric oxide donor andrographolide enhances humoral and cell-mediated immune responses. Cell. Mol. Biol..

[14] Su H., Mo J., Ni J., Ke H., Bao T., Xie J., Xu Y., Xie L., Chen W. (2020). Andrographolide exerts antihyperglycemic effect through strengthening intestinal barrier function and increasing microbial composition of *Akkermansia muciniphila*. Oxid. Med. Cell. Longev..

[15] Gupta S., Mishra K.P., Ganju L. (2017). Broad-spectrum antiviral properties of andrographolide. Archiv. Virol..

[16] Panraksa P., Ramphan S., Khongwichit S., Smith D.R. (2017). Activity of andrographolide against dengue virus. Antivir. Res..

[17] Shi T.H., Huang Y.L., Chen C.C., Pi W.C., Hsu Y.L., Lo L.C., Chen W.-Y., Fu S.-L., Lin C.H. (2020). Andrographolide and its fluorescent derivative inhibit the main proteases of 2019-nCoV and SARS-CoV through covalent linkage. Biochem. Biophys. Res. Commun..

[18] Srivastava N., Garg P., Srivastava P., Seth P.K. (2021). A molecular dynamics simulation study of the ACE2 receptor with screened natural inhibitors to identify novel drug candidate against COVID-19. PeerJ.

[19] Das S., Mishra K.P., Ganju L., Singh S.B. (2017). Andrographolide-A promising therapeutic agent, negatively regulates glial cell derived neurodegeneration of prefrontal cortex, hippocampus and working memory impairment. J. Neuroimmunol..

[20] Chen S., Luo Z., Chen X. (2020). Andrographolide mitigates cartilage damage via miR-27-3p-modulated matrix metalloproteinase13 repression. J. Gene Med..

[21] Zhang H., Li S., Si Y., Xu H. (2021). Andrographolide and its derivatives: Current achievements and future perspectives. Eur. J. Med. Chem..

[22] Lee K.-C., Chang H.-H., Chung Y.-H., Lee T.-Y. (2011). Andrographolide acts as an anti-inflammatory agent in LPS-stimulated RAW264. 7 macrophages by inhibiting STAT3-mediated suppression of the NF-κB pathway. J. Ethnopharmacol..

[23] Liu W., Fan T., Li M., Zhang G., Guo W., Yang X., Jiang C., Li X., Xu X., Tang A. (2020). Andrographolide potentiates PD-1 blockade immunotherapy by inhibiting COX2-mediated PGE2 release. Int. Immunopharmacol..

[24] Yuan L., Zhang C., Sun H., Liu Q., Huang J., Sheng L., Lin B., Wang J., Chen L. (2016). The semi-synthesis of novel andrographolide analogues and anti-influenza virus activity evaluation of their derivatives. Bioorg. Med. Chem. Lett..

[25] Wang W., Wu Y., Chen X., Zhang P., Li H., Chen L. (2019). Synthesis of new ent-labdane diterpene derivatives from andrographolide and evaluation of their anti-inflammatory activities. Eur. J. Med. Chem..

[26] Jiang M., Sheng F., Zhang Z., Ma X., Gao T., Fu C., Li P. (2021). *Andrographis paniculata* (Burm.f.) Nees and its major constituent andrographolide as potential antiviral agents. J. Ethnopharmacol..

[27] Tan W.S.D., Liao W., Zhou S., Wong W.F. (2017). Is there a future for andrographolide to be an anti-inflammatory drug? Deciphering its major mechanisms of action. Biochem. Pharmacol..

[28] Uthirapandi V., Subramanian S.R., Ponnerulan B., Saminathan E., Narayanan V., Durairaj K. (2021). Andrographolide production and enhanced antioxidant activity in *Andrographis paniculata* (Burm f.) Nees. promoted by seaweed liquid extracts. Braz. J. Bot..

[29] Lin H.C., Lii C.K., Chen H.C., Lin A.H., Yang Y.C., Chen H.W. (2018). Andrographolide inhibits oxidized LDL-induced cholesterol accumulation and foam cell formation in macrophages. Am. J. Chin. Med..

[30] Hossain S., Urbi Z., Karuniawati H., Mohiuddin R.B., Moh Qrimida A., Allzrag A.M.M., Ming L.C., Pagano E., Capasso R. (2021). *Andrographis paniculata* (Burm. F.) wall. Ex Nees: An updated review of phytochemistry, antimicrobial-pharmacology, and clinical safety and efficacy. Life.

[31] Kumar S., Singh B., Bajpai V. (2021). *Andrographis paniculata* (Burm.f.) Nees: Traditional uses, phytochemistry, pharmacological properties and quality control/quality assurance. J. Ethnopharmacol..

[32] Gorter M. (1911). The bitter constituent of *Andrographis paniculata* Nees. Rec. Trav. Chim..

[33] Kumar G., Singh D., Tali J.A., Dheer D., Shankar R. (2020). Andrographolide: Chemical modification and its effect on biological activities. Bioorg. Chem..

[34] Rafi M., Karomah A.H., Heryanto R., Septaningsih D.A., Kusuma W.A., Amran M.B., Rohman A., Prajogo B. (2022). Metabolite profiling of *Andrographis paniculata* leaves and stem extract using UHPLC-Orbitrap-MS/MS. Nat. Prod. Res..

[35] Bhat M.A., Murthy H.N. (2021). Isolation of andrographolide from *Andrographis lineata* Wall. ex Nees var. lawii C.B. clarke and its anticancer activity against human ovarian teratocarcinoma. Pharmacog. J..

[36] Zeng B., Wei A., Zhou Q., Yuan M., Lei K., Liu Y., Song J., Guo L., Ye Q. (2022). Andrographolide: A review of its pharmacology, pharmacokinetics, toxicity and clinical trials and pharmaceutical researches. Phytother. Res..

[37] Jayakumar T., Hsieh C.-Y., Lee J.-J., Sheu J.-R. (2013). Experimental and clinical pharmacology of *Andrographis paniculata* and its major bioactive phytoconstituent andrographolide. Evid. Based Complement Alternat. Med..

[38] Islam M.T., Ali E.S., Uddin S.J., Islam M.A., Shaw S., Khan I.N., Saravi S.S.S., Ahmad S., Rehman S., Gupta V.K. (2018). Andrographolide, a diterpene lactone from *Andrographis paniculata* and its therapeutic promises in cancer. Cancer Lett..

[39] Kandanur S.G.S., Tamang N., Golakoti N.R., Nanduri S. (2019). Andrographolide: A natural product template for the generation of structurally and biologically diverse diterpenes. Eur. J. Med. Chem..

[40] Kim T.G., Hwi K.K., Hung C.S. (2005). Morphological and biochemical changes of andrographolide-induced cell death in human prostatic adenocarcinoma PC-3 cells. In Vivo.

[41] Yang S., Evens A.M., Prachand S., Singh A.T.K., Bhalla S., David K., Gordon L.I. (2010). Mitochondrial-mediated apoptosis in lymphoma cells by the diterpenoid lactone andrographolide, the active component of *Andrographis paniculata*. Clin. Cancer Res..

[42] Yuwen D., Mi S., Ma Y., Guo W., Xu Q., Shen Y., Shu Y. (2017). Andrographolide enhances cisplatin-mediated anticancer effects in lung cancer cells through blockade of autophagy. Anti-Cancer Drugs.

[43] Devendar P., Nayak V.L., Yadav D.K., Kumar A.N., Kumar J.K., Srinivas K.S., Sridhar B., Khan F., Sastry K.P., Ramakrishna S. (2015). Synthesis and evaluation of anticancer activity of novel andrographolide derivatives. MedChemComm.

[44] Lim J.C.W., Jeyaraj E.J., Sagineedu S.R., Wong W.S.F., Stanslas J. (2015). SRS06, a new semisynthetic andrographolide derivative with improved anticancer potency and selectivity, inhibits nuclear factor-κB nuclear binding in the A549 non-small cell lung cancer cell line. Pharmacology.

[45] Peng Y., Li J., Sun Y., Chan J.Y.-W., Sheng D., Wang K., Wei P., Ouyang P., Wang D., Lee S.M.Y. (2015). SAR studies of 3, 14, 19-derivatives of andrographolide on anti-proliferative activity to cancer cells and toxicity to zebrafish: An in vitro and in vivo study. RSC Adv..

[46] Wanandi S.I., Limanto A., Yunita E., Syahrani R.A., Louisa M., Wibowo A.E., Arumsari S. (2020). In silico and in vitro studies on the anti-cancer activity of andrographolide targeting survivin in human breast cancer stem cells. PLoS ONE.

[47] Luo W., Jia L., Zhang J.W., Wang D.J., Ren Q., Zhang W. (2021). Andrographolide against lung cancer-new pharmacological insights based on high-throughput metabolomics analysis combined with network pharmacology. Front. Pharmacol..

[48] Vukmirovic D., Vo N.T.K., Seymour C., Rollo D., Mothersill C. (2021). Influence of common dietary supplements (curcumin, andrographolide, and d-limonene) on the radiobiological responses of p53-competent colonic cancer epithelial cells. Int. J. Radiation Biol..

[49] Banerjee V., Sharda N., Huse J., Singh D., Sokolov D., Czinn S.J., Blanchard T.G., Banerjee A. (2021). Synergistic potential of dual andrographolide and melatonin targeting of metastatic colon cancer cells: Using the Chou-Talalay combination index method. Eur. J. Pharmacol..

[50] Bi R., Deng Y.Y., Tang C., Xuan L., Xu B., Du Y.J., Wang C., Wei W. (2020). Andrographolide sensitizes human renal carcinoma cells to TRAILinduced apoptosis through upregulation of death receptor 4. Oncol. Rep..

[51] Pasha A., Kumbhakar D.V., Doneti R., Kumar K., Dharmapuri G., Poleboyina P.K., Heena S.K., Basavaraju P., Pasumarthi D., Annapurna S.D. (2021). Inhibition of inducible nitric oxide synthase (iNOS) by andrographolide and in vitro evaluation of its antiproliferative and proapoptotic effects on cervical cancer. Oxid. Med. Cell. Longev..

[52] Shi L., Zhang G.Q., Zheng Z.Y., Lu B., Ji L.L. (2017). Andrographolide reduced VEGFA expression in hepatoma cancer cells by inactivating HIF-1 alpha: The involvement of JNK and MTA1/HDCA. Chem. Biol. Interact..

[53] Banerjee M., Chattopadhyay S., Choudhuri T., Bera R., Kumar S., Chakraborty B., Mukherjee S.K. (2016). Cytotoxicity and cell cycle arrest induced by andrographolide lead to programmed cell death of MDA-MB-231 breast cancer cell line. J. Biomed. Sci..

[54] Dai L., Wang G., Pan W. (2017). Andrographolide inhibits proliferation and metastasis of SGC7901 gastric cancer cells. Biomed. Res. Int..

[55] Khan I., Khan F., Farooqui A., Ansari I.A. (2018). Andrographolide exhibits anticancer potential against human colon cancer cells by inducing cell cycle arrest and programmed cell death via augmentation of intracellular reactive oxygen species level. Nutr. Cancer.

[56] Chen W., Feng L., Nie H., Zheng X. (2012). Andrographolide induces autophagic cell death in human liver cancer cells through cyclophilin D-mediated mitochondrial permeability transition pore. Carcinogenesis.

[57] Das S., Rahaman A., Nath R., Talukdar A.D., Nath D., Bhattacharjee S., Mandal D.P., Choudhury M.D., Das D., Das G. (2023). Effect of acetone fraction of *Ottelia alismoides* on the G2/M cell cycle arrest and apoptosis in the human carcinoma cell lines. J. Ethnopharmacol..

[58] Xie J., Peng L.-J., Yang M.-R., Jiang W.-W., Mao J.-Y., Shi C.-Y., Tian Y., Sheng J. (2021). Alkaloid extract of *Moringa oleifera* lam. Exerts antitumor activity in human non-small-cell lung cancer via modulation of the JAK2/STAT3 signaling pathway. Evid. Based Complement. Altern. Med..

[59] Saqr A.A., Khafagy E.-S., Aldawsari M.F., Almansour K., Abu Lila A.S. (2022). Screening of apoptosis pathway-mediated anti-proliferative activity of the phytochemical compound furanodienone against human non-small lung cancer A-549 cells. Life.

[60] Sheeja K., Kuttan G. (2007). Activation of cytotoxic T lymphocyte responses and attenuation of tumor growth in vivo by *Andrographis paniculata* extract and andrographolide. Immunopharmacol. Immunotoxicol..

[61] Cheung H.-Y., Cheung S.-H., Li J., Cheung C.-S., Lai W.-P., Fong W.-F., Leung F.-M. (2005). Andrographolide isolated from *Andrographis paniculata* induces cell cycle arrest and mitochondrial-mediated apoptosis in human leukemic HL-60 cells. Planta Med..

[62] Kim Y.S., Milner J.A. (2005). Targets for indole-3-carbinol in cancer prevention. J. Nutr. Biochem..

[63] Sukumari-Ramesh S., Bentley J.N., Laird M.D., Singh N., Vender J.R., Dhandapani K.M. (2011). Dietary phytochemicals induce p53-and caspase-independent cell death in human neuroblastoma cells. Int. J. Develop. Neurosci..

[64] Wu Y., Zhou B.P. (2009). Inflammation: A driving force speeds cancer metastasis. Cell Cycle.

[65] Zhou J., Lu G.-D., Ong C.-S., Ong C.-N., Shen H.-M. (2008). Andrographolide sensitizes cancer cells to TRAIL-induced apoptosis via p53-mediated death receptor 4 up-regulation. Mol. Cancer Ther..

[66] Chun J.Y., Tummala R., Nadiminty N., Lou W., Liu C., Yang J., Evans C.P., Zhou Q., Gao A.C. (2010). Andrographolide, an herbal medicine, inhibits interleukin-6 expression and suppresses prostate cancer cell growth. Genes Cancer.

[67] Shi M.D., Lin H.H., Lee Y.C., Chao J.K., Lin R.A., Chen J.H. (2008). Inhibition of cell-cycle progression in human colorectal carcinoma Lovo cells by andrographolide. Chem. Biol. Interact..

[68] Satyanarayana C., Deevi D.S., Rajagopalan R., Srinivas N., Rajagopal S. (2004). DRF 3188 a novel semi-synthetic analog of an-drographolide: Cellular response to MCF 7 breast cancer cells. BMC Cancer.

[69] Rajagopal S., Kumar R.A., Deevi D.S., Satyanarayana C., Rajagopalan R. (2003). Andrographolide, a potential cancer therapeutic agent isolated from *Andrographis paniculate*. J. Exp. Therapeut. Oncol..

[70] Yan J., Chen Y., He C., Yang Z.-Z., Lü C., Chen X.-S. (2012). Andrographolide induces cell cycle arrest and apoptosis in human rheumatoid arthritis fibroblast-like synoviocytes. Cell Biol. Toxicol..

[71] Roy P., Das S., Mondal A., Chatterji U., Mukherjee A. (2012). Nanoparticle engineering enhances anticancer efficacy of andrographolide in MCF-7 cells and mice bearing EAC. Curr. Pharm. Biotechnol..

[72] Kumar S., Patil H.S., Sharma P., Kumar D., Dasari S., Puranik V.G., Thulasiram H.V., Kundu G.C. (2012). Andrographolide inhibits osteopontin expression and breast tumor growth through down regulation of PI3 kinase/Akt signaling pathway. Curr. Mol. Med..

[73] Zhang Q.-Q., Zhou D.-L., Ding Y., Liu H.-Y., Lei Y., Fang H.-Y., Yang Y. (2014). Andrographolide inhibits melanoma tumor growth by inactivating the TLR4/NF-κB signaling pathway. Melanoma Res..

[74] Wong C.C., Lim S.H., Sagineedu S.R., Lajis N.H., Stanslas J. (2016). SRJ09, a promising anticancer drug lead: Elucidation of mechanisms of antiproliferative and apoptogenic effects and assessment of in vivo antitumor efficacy. Pharmacol. Res..

[75] Sheeja K., Guruvayoorappan C., Kuttan G. (2007). Antiangiogenic activity of *Andrographis paniculata* extract and andrographolide. Int. Immunopharmacol..

[76] Wang S., Li H., Chen S., Wang Z., Yao Y., Chen T., Ye Z., Lin P. (2020). Andrographolide induces apoptosis in human osteosarcoma cells via the ROS/JNK pathway. Int. J. Oncol..

[77] Chao H.-P., Kuo C.-D., Chiu J.-H., Fu S.-L. (2010). Andrographolide exhibits anti-invasive activity against colon cancer cells via inhibition of MMP2 activity. Planta Med..

[78] Lin H.-H., Tsai C.-W., Chou F.-P., Wang C.-J., Hsuan S.-W., Wang C.-K., Chen J.-H. (2011). Andrographolide down-regulates hypoxia-inducible factor-1α in human non-small cell lung cancer A549 cells. Toxicol. Appl. Pharmacol..

[79] Xiao X.-W., Fu H.-Z., Luo Y.-H., Wei X.-Y. (2013). Potential anti-angiogenic sulfates of andrographolide. J. Asian Nat. Prod. Res..

[80] Kajal K., Panda A.K., Bhat J., Chakraborty D., Bose S., Bhattacharjee P., Sarkar T., Chatterjee S., Kar S.K., Sa G. (2019). Andrographolide binds to ATP-binding pocket of VEGFR2 to impede VEGFA-mediated tumor-angiogenesis. Sci. Rep..

[81] Dai J., Lin Y., Duan Y., Li Z., Zhou D., Chen W., Wang L., Zhang Q.Q. (2017). Andrographolide Inhibits Angiogenesis by Inhibiting the Mir-21-5p/TIMP3 Signaling Pathway. Int. J. Biol. Sci..

[82] Yadav R.V., Sadhukhan S., Saha M.L., Ghosh S., Das M. (2022). Exploring the mechanism of andrographolide in the treatment of gastric cancer through network pharmacology and molecular docking. Sci. Rep..

[83] Li J., Li F., Tang F., Zhang J., Li R., Sheng D., Lee S.M.Y., Zhou G.C., Leung G.P.H. (2020). AGS-30, an andrographolide derivative, suppresses tumor angiogenesis and growth in vitro and in vivo. Biochem. Pharmacol..

[84] Udomwan P., Pientong C., Tongchai P., Burassakarn A., Sunthamala N., Roytrakul S., Suebsasana S., Ekalaksananan T. (2021). Proteomics analysis of andrographolide-induced apoptosis via the regulation of tumor suppressor p53 proteolysis in cervical cancer-derived human papillomavirus 16-positive cell lines. Int. J. Mol. Sci..

[85] Tohkayomatee R., Reabroi S., Tungmunnithum D., Parichatikanond W., Pinthong D. (2022). Andrographolide exhibits anticancer activity against breast cancer cells (MCF-7 and MDA-MB-231 Cells) through suppressing cell proliferation and inducing cell apoptosis via inactivation of ER-α receptor and PI3K/AKT/mTOR signaling. Molecules.

[86] Doi H., Matsui T., Dijkstra J.M., Ogasawara A., Higashimoto Y., Imamura S., Ohye T., Takematsu H., Katsuda I., Akiyama H. (2022). Andrographolide, isolated from *Andrographis paniculata*, induces apoptosis in monocytic leukemia and multiple myeloma cells via augmentation of reactive oxygen species production. F1000Research.

[87] Liu G., Chu H. (2018). Andrographolide inhibits proliferation and induces cell cycle arrest and apoptosis in human melanoma cells. Oncol. Lett..

[88] He C., Klionsky D.J. (2009). Regulation mechanisms and signaling pathways of autophagy. Ann. Rev. Genet..

[89] Codogno P., Meijer A.J. (2005). Autophagy and signaling: Their role in cell survival and cell death. Cell Death Differ..

[90] Azad M.B., Chen Y., Gibson S.B. (2009). Regulation of autophagy by reactive oxygen species (ROS): Implications for cancer progression and treatment. Antiox. Redox. Signal.

[91] Corcelle E., Djerbi N., Mari M., Nebout M., Fiorini C., Fenichel P., Hofman P., Poujeol P., Mograbi B. (2007). Control of the autophagy maturation step by the MAPK ERK and p38: Lessons from environmental carcinogens. Autophagy.

[92] He Z.J., Zhu F.Y., Li S.S., Zhong L., Tan H.Y., Wang K. (2017). Inhibiting ROS-NF-kappaB-dependent autophagy enhanced brazilin-induced apoptosis in head and neck squamous cell carcinoma. Food Chem. Toxicol..

[93] Heras-Sandoval D., Perez-Rojas J.M., Hernandez-Damian J., Pedraza-Chaverri J. (2014). The role of PI3K/AKT/mTOR pathway in the modulation of autophagy and the clearance of protein aggregates in neurodegeneration. Cell Signal.

[94] Liu Y., Zhang Y., Zou J., Yan L., Yu X., Lu P., Wu X., Li Q., Gu R., Zhu D. (2017). Andrographolide induces autophagic cell death and inhibits invasion and metastasis of human osteosarcoma cells in an autophagy-dependent manner. Cell Physiol. BioChem.

[95] Krithika R., Verma R.J., Shrivastav P.S. (2013). Antioxidative and cytoprotective effects of andrographolide against CCl4-induced hepatotoxicity in HepG2 cells. Hum. Exp. Toxicol..

[96] Shen Y.-C., Chen C.-F., Chiou W.-F. (2002). Andrographolide prevents oxygen radical production by human neutrophils: Possible mechanism(s) involved in its anti-inflammatory effect. Br. J. Pharmacol..

[97] Peng S., Gao J., Liu W., Jiang C., Yang X., Sun Y., Guo W., Xu Q. (2016). Andrographolide ameliorates OVA-induced lung injury in mice by suppressing ROS-mediated NF- B signaling and NLRP3 inflammasome activation. Oncotarget.

[98] Zhan J.Y.-X., Wang X.-F., Liu Y.-H., Zhang Z.-B., Wang L., Chen J.-N., Huang S., Zeng H.-F., Lai X.-P. (2016). Andrographolide sodium bisulfate prevents UV-induced skin photoaging through inhibiting oxidative stress and inflammation. Mediat. Inflamm..

[99] Thangathirupathi A., Ali N., Natarajan P., Ramesh Kumar D. (2013). Molecular docking studies of andrographolide with xanthine oxidase. Asian J. Pharm. Clin. Res..

[100] Rahmi E.P., Kumolosasi E., Jalil J., Buang F., Jamal J.A. (2022). Extracts of *Andrographis paniculata* (Burm.f.) nees leaves exert anti-gout effects by lowering uric acid levels and reducing monosodium urate crystalinduced inflammation. Front. Pharmacol..

[101] Chern C.-M., Liou K.-T., Wang Y.-H., Liao J.-F., Yen J.-C., Shen Y.-C. (2011). Andrographolide inhibits PI3K/AKT-dependent NOX2 and iNOS expression protecting mice against hypoxia/ischemia-induced oxidative brain injury. Planta Med..

[102] Liang E., Liu X., Du Z., Yang R., Zhao Y. (2018). Andrographolide ameliorates diabetic cardiomyopathy in mice by blockage of oxidative damage and NF-B-mediated inflammation. Oxid. Med. Cell Longev..

[103] Geng J., Liu W., Xiong Y., Ding H., Jiang C., Yang X., Li X., Elgehama A., Sun Y., Xu Q. (2018). Andrographolide sulfonate improves Alzheimer-associated phenotypes and mitochondrial dysfunction in APP/PS1 transgenic mice. Biomed. Pharmacother..

[104] Das S., Gautam N., Dey S.K., Maiti T., Roy S. (2009). Oxidative stress in the brain of nicotine-induced toxicity: Protective role of *Andrographis paniculata* Nees and vitamin E. Appl. Physiol. Nutr. Metab..

[105] Wong D.P.W., Ng M.Y., Leung J.Y., Boh B.K., Lim E.C., Tan S.H., Lim S., Seah W.H., Hu C.Z., Ho B.C. (2018). Regulation of the NRF2 transcription factor by andrographolide and organic extracts from plant endophytes. PLoS ONE.

[106] Fu K., Chen H., Wang Z., Cao R. (2021). Andrographolide attenuates inflammatory response induced by LPS via activating Nrf2 signaling pathway in bovine endometrial epithelial cells. Res. Vet. Sci..

[107] Zhang C., Qiu X. (2015). Andrographolide radiosensitizes human ovarian cancer SKOV3 xenografts due to an enhanced apoptosis and autophagy. Tumour Biol..

[108] Chen D., Song Y., Lu Y., Xue X. (2013). Synthesis and in vitro cytotoxicity of andrographolide-19-oic acid analogues as anti-cancer agents. Bioorg. Med. Chem. Lett..

[109] Zhu Y.-Y., Yu G., Zhang Y., Xu Z., Wang Y.-Q., Yan G.-R., He Q.-Y. (2013). A novel andrographolide derivative AL-1 exerts its cytotoxicity on K562 cells through a ROS-dependent mechanism. Proteomics.

[110] Lu H., Zhang X.-Y., Wang Y.-Q., Zheng X.-L., Yin Z., Xing W.-M., Zhang Q. (2014). Andrographolide sodium bisulfate-induced apoptosis and autophagy in human proximal tubular endothelial cells is a ROS-mediated pathway. Environm. Toxicol. Pharmacol..

[111] Wang Z.M., Kang Y.H., Yang X., Wang J.F., Zhang Q., Yang B.X., Zhao K.L., Xu L.P., Yang L.P., Ma J.X. (2016). Andrographolide radiosensitizes human esophageal cancer cell line ECA109 to radiation in vitro. Dis. Esophagus.

[112] Hung S.K., Hung L.C., Kuo C.D., Lee K.Y., Lee M.S., Lin H.Y., Chen Y.J., Fu S.L. (2010). Andrographolide sensitizes Ras-transformed cells to radiation in vitro and in vivo. J. Radiat. Oncol. Biol. Phys..

[113] Yang P.-Y., Hsieh P.-L., Wang T.H., Yu C.-C., Lu M.-Y., Liao Y.-W., Lee T.-H., Peng C.-Y. (2017). Andrographolide impedes cancer stemness and enhances radio-sensitivity in oral carcinomas via miR-218 activation. Oncotarget.

[114] Li X., Tian R., Liu L., Wang L., He D., Cao K., Ma J.K., Huang C. (2020). Andrographolide enhanced radiosensitivity by downregulating glycolysis via the inhibition of the PI3K-Akt-mTOR signaling pathway in HCT116 colorectal cancer cells. J. Int. Med. Res..

[115] Zhang D., Huang Y., Qiao Y., Xia C., Luo Y., Chen Z. (2016). Antibacterial activity of inclusion complexes of andrographolide and 14-acetylandrographolide by hydroxypropyl-β-cyclodextrin. J. Nanjing Agric. Univ..

[116] Yuan M., Meng W., Liao W., Lian S. (2018). Andrographolide Antagonizes TNF-α-Induced IL-8 via Inhibition of NADPH Oxidase/ROS/NF-κB and Src/MAPKs/AP-1 Axis in Human Colorectal Cancer HCT116 Cells. J. Agric. Food Chem..

[117] Farooqi A.A., Attar R., Sabitaliyevich U.Y., Alaaeddine N., de Sousa D.P., Xu B., Cho W.C. (2020). The prowess of andrographolide as a natural weapon in the war against cancer. Cancers.

[118] Zhou J., Ong C.-N., Hur G.-M., Shen H.-M. (2010). Inhibition of the JAK-STAT3 pathway by andrographolide enhances chemosensitivity of cancer cells to doxorubicin. Biochem. Pharmacol..

[119] Bao G.-Q., Shen B.-Y., Pan C.-P., Zhang Y.-J., Shi M.-M., Peng C.-H. (2013). Andrographolide causes apoptosis via inactivation of STAT3 and Akt and potentiates antitumor activity of gemcitabine in pancreatic cancer. Toxicol. Lett..

[120] Kotakadi V.S., Gaddam S.A., Rao Y.S., Prasad T., Reddy A.V., Gopal D.S. (2014). Biofabrication of silver nanoparticles using *Andrographis paniculata*. Eur. J. Med. Chem..

[121] Wang Z., He R., Chen Y., Wu F. (2016). Regio-selective PEGylation of 14-deoxy-11, 12-didehydroandrographolide and their biological evaluation. Tetrahedron.

[122] Bera R., Ahmed S.K., Sarkar L., Sen T., Karmakar S. (2014). Pharmacokinetic analysis and tissue distribution of andrographolide in rat by a validated LC-MS/MS method. Pharm. Biol..

[123] Balap A., Lohidasan S., Sinnathambi A., Mahadik K. (2017). Pharmacokinetic and pharmacodynamic interaction of andrographolide and standardized extract of *Andrographis paniculata* (Nees) with nabumetone in Wistar rats. Phytother. Res..

[124] Ye L., Wang T., Tang L., Liu W., Yang Z., Zhou J., Liu Z. (2011). Poor oral bioavailability of a promising anticancer agent andrographolide is due to extensive metabolism and efflux by P-glycoprotein. J. Pharm. Sci..

[125] Balap A., Lohidasan S., Sinnathambi A., Mahadik K. (2017). Herb-drug interaction of *Andrographis paniculata* (Nees) extract and andrographolide on pharmacokinetic and pharmacodynamic of naproxen in rats. J. Ethnopharmacol..

[126] Zhang S.-Q., Wang X., Zhang Y., Li X. (2020). Pharmacokinetics of andrographolide sodium bisulphite and its related substance in rats by liquid chromatography–tandem mass spectrometry. J. Anal. Chem..

[127] Zhang S.Q., Fan Y.M. (2012). Determination of andrograpolide sodium bisulphite in Beagle dog plasma by LC-MS/MS and its application to pharmacokinetics. J. Chromatogr. B.

[128] Godugu D., Rupula K., Sashidhar R.B. (2018). Binding studies of andrographolide with human serum albumin: Molecular docking, chromatographic and spectroscopic studies. Protein Pept. Lett..

[129] Zhao H.-Y., Hu H., Wang Y.-T. (2013). Comparative metabolism and stability of andrographolide in liver microsomes from humans, dogs and rats using ultra-performance liquid chromatography coupled with triple-quadrupole and Fourier transform ion cyclotron resonance mass spectrometry. Rapid Commun. Mass Spectrom..

[130] Yu Z., Chen Z., Li Q., Yang K., Huang Z., Wang W., Hu H. (2021). What dominates the changeable pharmacokinetics of natural sesquiterpene lactones and diterpene lactones: A review focusing on absorption and metabolism. Drug Met. Rev..

[131] Panossian A., Hovhannisyan A., Mamikonyan G., Abrahamian H., Hambardzumyan E., Gabrielian E., Goukasova G., Wikman G., Wagner H. (2000). Pharmacokinetic and oral of andrographolide from *Andrographis paniculata* fixed combination Kan Jang in rats and human. Phytomedicine.

[132] Qiu F., Cui L., Chen L., Sun J., Yao X. (2012). Two novel creatinine adducts of andrographolide in human urine. Xenobiotica.

[133] Zhang X., Zhang X., Wang X., Zhao M. (2018). Influence of andrographolide on the pharmacokinetics of warfarin in rats. Pharm. Biol..

[134] Suriyo T., Chotirat S., Rangkadilok N., Pholphana N., Satayavivad J. (2021). Interactive effects of *Andrographis paniculata* extracts and cancer chemotherapeutic 5-Fluorouracil on cytochrome P450s expression in human hepatocellular carcinoma HepG2 cells. J. Herb. Med..

[135] Pholphana N., Panomvana D., Rangkadilok N., Suriyo T., Puranajoti P., Ungtrakul T., Pongpun W., Thaeopattha S., Songvut P., Satayavivad J. (2016). Andrographis paniculata: Dissolution investigation and pharmacokinetic studies of four major active diterpenoids after multiple oral dose administration in healthy Thai volunteers. J. Ethnopharmacol..

[136] Gu L.L., Zhang X.Y., Xing W.M., Xu J.D., Lu H. (2016). Andrographolide-induced apoptosis in human renal tubular epithelial cells: Roles of endoplasmic reticulum stress and inflammatory response. Env. Toxicol. Pharmacol..

[137] Liang H.X., Lu S.S., Yan Z., Kuang Y.P., Zhu X.X., Yan Z.G., Lyu Q.F. (2017). Andrographolide disrupts meiotic maturation by blocking cytoskeletal reorganisation and decreases the fertilisation potential of mouse oocytes. Reprod. Fertil. Dev..

[138] Ciampi E., Uribe-San-Martin R., Carcamo C., Cruz J.P., Reyes A., Reyes D., Hancke J. (2020). Efficacy of andrographolide in not active progressive multiple sclerosis: A prospective exploratory double-blind, parallel-group, randomized, placebo-controlled trial. BMC Neurol..

[139] Calabrese C., Berman S.H., Babish J.G., Ma X., Shinto L., Dorr M., Standish L.J. (2000). A phase I trial of andrographolide in HIV positive patients and normal volunteers. Phytother. Res..

[140] Chandrama Singh S., Choudhary M., Mourya A., Khatri D.K., Singh P.K., Madan J., Singh H. (2022). Acute and subacute toxicity assessment of andrographolide-2-hydroxypropyl-β-cyclodextrin complex via oral and inhalation route of administration in sprague-dawley rats. Sci. World J..

[141] Sato H., Hiraki M., Namba T., Egawa N., Baba K., Tanaka T., Noshiro H. (2018). Andrographolide induces degradation of mutant p53 via activation of Hsp70. Int. J. Oncol..

[142] Zhang H.T., Yang J., Liang G.H., Gao X.J., Sang Y., Gui T., Lian Z.J., Tam M.S., Zha Z.G. (2017). Andrographolide induces cell cycle arrest and apoptosis of Chondrosarcoma by targeting TCF-1/SOX9 Axis. J. Cell. Biochem..

